# Analysis of the Sam50 translocase of Excavate organisms supports evolution of divergent organelles from a common endosymbiotic event

**DOI:** 10.1042/BSR20130049

**Published:** 2013-12-03

**Authors:** Christopher J. Kay, Karen Lawler, Ian D. Kerr

**Affiliations:** *School of Life Sciences, University of Nottingham Medical School, Queen's Medical Centre, Nottingham NG7 2UH, U.K.

**Keywords:** evolution, hydrogenosome, membrane, organelles, *Trichomonas*, β-barrel, aa, amino acids, BN-PAGE, Blue native-polyacrylamide gel electrophoresis, CDD, conserved domains database, DSP, dithiobis(succinimidyl propionate), DTT, dithiothreitol, EST, Expressed Sequence Tag, HA, haemaglutinin, HMM, hidden Markov model, MBS, maleimidobenzoyl-hydroxysuccinimide ester, OEP, outer envelope protein, OMP, outer membrane protein, POTRA, polypeptide-transport-associated domain, SAM, sorting and assembly machinery, TBS-T, TBS with 0.1% (v/v) Tween, TOC, translocon of the outer membrane of the chloroplast, TOM, translocase of the outer membrane

## Abstract

As free-living organisms the ancestors of mitochondria and plastids encoded complete genomes, proteomes and metabolomes. As these symbionts became organelles all these aspects were reduced – genomes have degenerated with the host nucleus now encoding the most of the remaining endosymbiont proteome, while the metabolic processes of the symbiont have been streamlined to the functions of the emerging organelle. By contrast, the topology of the endosymbiont membrane has been preserved, necessitating the development of complex pathways for membrane insertion and translocation. In this study, we examine the characteristics of the endosymbiont-derived β-barrel insertase Sam50^1^ in the excavate super-group. A candidate is further characterized in *Trichomonas vaginalis*, an unusual eukaryote possessing degenerate hydrogen-producing mitochondria called hydrogenosomes. This information supports a mitochondriate eukaryotic common ancestor with a similarly evolved β-barrel insertase, which has continued to be conserved in degenerate mitochondria.

## INTRODUCTION

Organellogenesis is dominated by evolutionary processes where the nucleus establishes genetic primacy over the endosymbiont genome. This process need not begin with eukaryotic proteins being inserted into endosymbiont membranes, although it has been proposed [1], but this is ultimately essential. The emergent multi-membrane translocase systems of organelles are the products of an incremental evolutionary process drawing on both prokaryotic conservation and eukaryotic innovation [2]. This study specifically considers the mitochondrion, an organelle with two bounding membranes descended from an α-proteobacterial ancestor [3]. The outer mitochondrial membrane presents the first barrier to cytosolically translated proteins, and simultaneously an early evolutionary obstacle to organellogenesis. In some proposed timelines, nuclear control was first established over the outer membrane, and incrementally towards the interior [2]. Thus, the oldest processes in the transformation of endosymbiont to organelle should be revealed by studying the outer membrane system.

The degeneration of the endosymbiont genome necessitated increasing nuclear control to maintain mitochondrial viability. To communicate through the outer membrane, the nuclear genome evolved to encode two essential membrane translocase complexes, namely a gateway complex [TOM (translocase of the outer membrane,)] and a membrane protein insertase complex [SAM (sorting and assembly machinery)]. The core translocases of both complexes owe their heritage to prokaryotic proteins, and while both are predicted to function as β-barrels, the translocases of SAM and TOM complexes are of distinct descent [4]. The SAM complex translocase, originated within the mitochondrial endosymbiont as a bacterial OMP (outer membrane protein) 85-like insertase, and served to initially insert endosymbiont and imported nuclear proteins into the outer membrane. Once acquired by the nucleus, Sam50 would ultimately replace the endogenous Omp85 during endosymbiont genome degradation [2,5], thereby securing nuclear control over the mitochondrial outer membrane.

In this study, the excavate super-group is examined for Sam50 homologues. The excavates have been suggested to be a monophyletic eukaryotic super-group, whose biology potentially illustrates features of ancient eukaryotes [6]. Many excavate species possess unusual metabolic organelles, which are now thought to be of mitochondrial descent [7], and demonstration of a shared outer membrane translocation machinery would further support this argument.

To date previous research has identified homologues to Sam50 in sequenced Trypanosomatids [8] and in *Trichomonas* [9]. By contrast, in the diplomonad Giardia, no homologue to Sam50 has been identified, although it possesses a homologue to the β-barrel Tom40 [10]. To further expand this knowledge, we use bioinformatics to examine the distribution and phylogeny of Sam50 in fully sequenced excavates as well as in preliminary data from *Euglena*, *Jakoba*, *Malawimonas*, *Reclimonas* and *Seculamonas*. In addition, we characterize the identified Sam50 homologue of *Trichomonas vaginalis* [9], providing evidence that it is a component of a membrane-associated complex in the hydrogenosome, consistent with a functional role in protein translocation.

## MATERIALS AND METHODS

### Production of HA (haemaglutinin)-tagged T.v.Sam50 transformant *T. vaginalis*

*T. vaginalis* strains ATCC30001 (C1) and G3 were maintained in the Diamond's medium supplemented with iron [11]. gDNA (genomic DNA) was prepared as described previously [12] and used to amplify T.v.Sam50 (primers, forward 5′-GACTCCCA-TATGTCATCTGCACCAGAGTGGTTC-3′ and reverse 5′-GA-GTCCCTCGAGAGCAGGAGTAATTCCGAGCTGATA-3′,) from *T. vaginalis* strain G3. PCR products were ligated C-terminal to a double haemaglutinin tag (HA–HA) in a modified pTagVag vector [13] by restriction sub-cloning. *T. vaginalis* strain C1 was then transfected by electroporation as previously described [12] and maintained in selective media containing G418.

### Subcellular fractionation of *T. vaginalis*

Transformant cultures expressing HA-tagged protein were grown to high density before cells were pelleted by centrifugation (1500 ***g***, 10 min, 4°C) and washed with SH buffer (250 mM sucrose, 20 mM Hepes)+10 mM β-mercaptoethanol, pelleted and resuspended 700 μl/g of cell pellet in SH+5 mM DTT (dithiothreitol) and protease inhibitors (TLCK; 25 μg/ml, leupeptin; 10 μg/ml). Cells were gently disrupted through needle passage (23-gauge) until greater than 90% of cells were lysed as determined by microscopy. This whole cell lysate was used to generate hydrogenosomal and cytosol fractions. First, cellular debris was pelleted through centrifugation (1000 ***g***, 5 min, 4°C) before a mixed organellar pellet was separated from the supernatant by centrifugation (8000 ***g***, 5 min, 4°C), crude cytosol was produced by clearing the supernatant twice at 16 000 ***g***, 15 min, 4°C. Hydrogenosomes were purified from the organellar pellet by density gradient ultracentrifugation in a separation medium of SH+protease inhibitors and Iodixanol at a concentration of 20–40%. Columns loaded with resuspended organelles were subject to centrifugation at 70 000 ***g*** for 2 h at 4°C. After separation the lower dark hydrogenosomal bands and lighter lysosomal bands were extracted [14], and diluted ten times in SH buffer. Purified organelles were collected at 8000×***g***, 10 min, 4°C and resuspended in SH+protease inhibitors+10% (v/v) glycerol before storage at −80°C. Organellar membranes were prepared from purified hydrogenosomes by sodium carbonate fractionation. Thawed organelles were resuspended in 1 mg/ml 0.1 sodium carbonate (pH 11.5) and incubated on ice for at least 90 min with periodic vortexing. Intact organelles were separated by centrifugation at 15 000 ***g*** for 15 min, before the membrane rafts were collected from the supernatant by ultracentrifugation at 100 000 ***g***, for 30 min at 4°C, soluble hydrogenosomal proteins were then precipitated from the supernatant by TCA (trichloroacetic acid) precipitation.

### Analysis of T.v.Sam50 protein complexes

The intermolecular interactions of T.v.Sam50 were examined through BN-PAGE (Blue native-polyacrylamide gel electrophoresis), chemical crosslinking and co-immunoprecipitation. For chemical crosslinking, purified hydrogenosomes were thawed and resuspended in 5 mg/ml SH buffer. Crosslinking reactions of 200 μl were performed with either DSP [dithiobis(succinimidyl propionate)] or MBS (maleimidobenzoyl-hydroxysuccinimide ester) crosslinking agents at a concentration of 0.5 mM. Reactions were quenched after 20 min by the addition of Tris pH 7.4 to a concentration of 10 mM. In reactions where DSP crosslinking was reversed DTT was added to the reaction to a final concentration of 0.1 M and incubated at 37°C for 5 min.

For BN-PAGE and co-immunoprecipitation hydrogenosomes were thawed, pelleted and resuspended in a solubilization buffer of composition 10% (v/v) glycerol, 250 mM NaCl, 50 mM Hepes, 5 mM MgCl_2_, 5 mM DTT, 5 mM EDTA and 1% (v/v) Roche protease inhibitor cocktail V at pH 7.4 and detergents DDM, Digitonin and Triton X-100, between 0.5 and 1.0% (v/v). Resuspended hydrogenosomes were rotated at 4°C for 90 min, and intact organelles and debris cleared from the solution by centrifugation (16 000×***g***, 4°C, 15 min). Cleared solubilized material was used for BN-PAGE, for co-immunoprecipitation. Triton X-100 solubilized samples were incubated overnight with 2 μl mouse monoclonal anti-HA antibody (Sigma) with rotation. Immunocomplexed proteins were extracted by the addition of 50 μl Protein-A and rotated at room temperature for a further hour. Non-specific proteins were washed from the beads with additional solubilization buffer (three washes of 20× bead volume) and bound immunocomplexed protein eluted with 50 μl 0.1 M glycine pH 2.1.

### Electrophoresis and Western blotting

Subcellular fractions, crosslinked and co-immunoprecipitated protein samples were separated on 1D (one-dimensional) SDS–PAGE gels carried out according to Laemmli [15] on 10–15% (w/v) gels. Detergent solubilized protein was mixed with a BN-PAGE sample buffer [5% (w/v) Coomassie Brilliant Blue G250, 0.75M 6-aminocaproic acid, 100 mM Bis-Tris, pH7.0] and was separated on 12% BN-PAGE gels as outlined by Schägger and von Jagow [16].

Electrophoresis gels were electro-blotted to PVDF membranes, which were then blocked with TBS-T [TBS with 0.1% (v/v) Tween] and 5% (w/v) non-fat dried skimmed milk powder, and subsequently incubated with primary antibody (mouse monoclonal anti-HA, 1:5000) for 1 h. Membranes were then washed with TBS-T, and incubated with goat anti-mouse-HRP (horseradish peroxidase)-conjugated secondary antibody (1:20000) in non-fat dried skimmed milk supplemented TBS-T for 1 h. Washed blots were then imaged using chemiluminescence (EZ-ECL; Geneflow) and imaged using a Fujifilm LAS-1000.

### Confocal microscopy

Transformant cells were collected from media by centrifugation and resuspended densely in PBS and deposited onto silane-coated slides (Sigma). Cells were covered and left to adhere for 30 min before excess were washed off with PBS. Cells were then fixed with 4% (w/v) paraformaldehyde with 0.1% (v/v) Triton X-100 for 20 min at room temperature. Fixed cells were washed with PBS before blocking with PBS supplemented with 0.25% (w/v) BSA and 0.25% (w/v) fish scale gelatin (blocking solution), primary antibody was then incubated in the same solution at 1:1000 for 1 h at room temperature before being washed twice with PBS before incubation of an Alexafluor 488 coupled secondary (1:1000) in blocking solution. Nuclei were stained with propidium iodide, cells were first treated with PBS supplemented with RNAase (100 μg/ml) at 37°C for 20 min to eliminate non-specific staining of RNA, and then with PBS with 3.3 μg/ml propidium iodide for 5 min. Excess nuclear stain was washed off with PBS before slides were mounted in PBS +50% (v/v) glycerol. Cells were imaged on a Zeiss 710 confocal laser scanning microscope. The Alexa tag was excited using a 488 nm argon laser and propidium iodide with a 562 nm solid-state laser. Light was collected on two seperate image channels (500–530 and 565–700 nm) to independently image each fluorophore. Confocal images were then further processed in Carl Zeiss Zen 2009 Light Edition and Carl Zeiss LSM Image Browser software.

### Bioinformatic analysis

Sequences for Sam50 and Omp85 homologues were collected from the Conserved Domains Database [17] from the entry Bac_surface_Ag giving an initial alignment of 70 sequences. Additional sequences were collected from NCBI's homologene for eukaryotic proteins of the Sam50, Toc (translocon of the outer membrane of the chloroplast) 75 and Oep80 families. HMM (hidden Markov models were constructed from alignments of collected proteins generated with MAFFT [18] and used to detect homologues in excavates using the HMMer web server search tool [19]. Recovered sequences were aligned with MAFFT with sequences from the CDD (conserved domains database) seed alignment and annotated reference sequences.

Dendrograms were constructed from aligned sequences using both a maximum-likelihood model and Bayesian analysis techniques. Maximum-likelihood trees were generated with PhyML [20] using a WAG rate matrix with estimated invariant and gamma models, and reproduced to 100 bootstraps. Bayesian analysis was performed using MrBayes [21] also with WAG rate matrix and estimated invariant and gamma models. Metropolis coupled Monte-Carlo was performed over an HMM chain length of 100000 with a sub-sampling frequency of 1 in 200. A 20% burn in was used on the collected trees and consensus trees assembled from the remaining outputs.

EST (Expressed Sequence Tag) libraries for incompletely sequenced protists were screened with tBLASTn using sequences collected from HMM searches. Aligned regions from sequences with E-values less than 1.0E-4 and sequence length >100 aa (amino acids) were assembled with reference sequences using ClustalW2 and MAFFT [22].

## RESULTS

### Putative Sam50-like insertases in excavates

Using reference sequences from CDD and previously annotated homologues to Sam50, an HMM was constructed, and was used with HMMer to detect putative homologues in *Leishmania*, *Naegleria gruberi*, *T. vaginalis* and *Trypanosoma* (see Supplementary Table S1 at http://www.bioscirep.org/bsr/033/bsr033e084add.htm)]. Our analysis detects the previously characterized sequences from *Trypanosoma brucei* (Trypanosoma DB Tb927.3.4380, Uniprot Q580K4) [8] and *Trichomonas* (TrichomonasDB TVAG_178100, Uniprot A2DIG2) [9]. Full-length protein sequences from the seed alignment and HMM results were used in tBLASTn to detect sequences from incompletely sequenced protists (Supplementary Table S1). These additional data suggest the potential for proteins containing N- and C-terminal fragments of Sam50-like proteins in the EST libraries of *Euglena*, *Jakoba*, *Malawimonas*, *Reclimonas* and *Seculamonas*. These analyses indicate the potential for Sam50-like proteins in most excavates for which there is good data. In exception to this, the genome-sequenced *Giardia lamblia* (synonymously *Giardia intestinalis*) was not detected to have any candidates, which is consistent with current research on giardial mitosome [23], neither was a candidate detected in the EST library of *Spironucleus*. To confirm that our HMM models were not merely identifying membrane β-barrel structures we constructed comparable models from reference sequence of TOC75 or OEP80 families. These HMMs failed to find any homologues in the excavate clade affording confidence in the specificity of these results. HMM models of OMP85 found a sequence in the incompletely sequenced *Trypanosoma congolense* (Supplementary Table S1); however, the protein identified is identical to a sequence in *Pseudomonas*, and likely to be an artefact of contamination.

### Consistent domain architecture in putative excavate insertases

**Figure 1:**
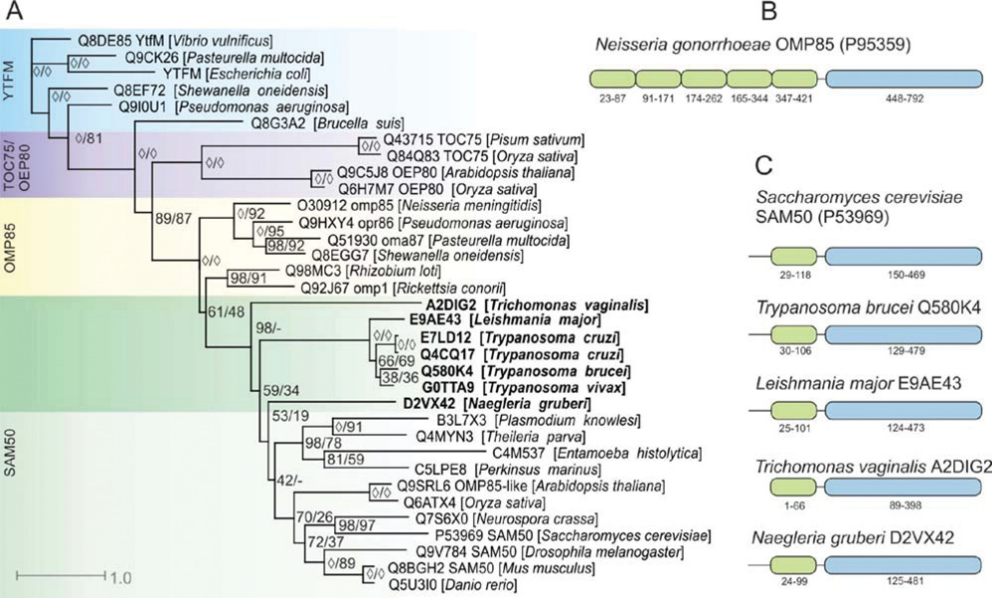
Outer membrane insertases are conserved in excavate species and share similar domain organization (**A**) Prokaryotic and eukaryotic sequences from the ‘bacterial surface antigen’ family in CDD were aligned with reference sequences of YtfM, BamA, Sam50, OEP 80 and Toc75 as well as Excavate candidate insertases. The resultant alignment was subject to Bayesian analysis with MrBayes, and distance (posterior probability) and boot strap values have been applied to a consensus dendrogram of 800 trees (left/first number, circles indicate >99% support), A maximum-likelihood analysis was also conducted using PhyML and repeated to 100 bootstraps, these support values are indicated in the second number, with ‘-’ indicating where there is disagreement in the consensus topology. Entries indicate their species, and Uniprot accession. (**B** and **C**) Candidate sequences detected from HMM searches were aligned with reference proteins, whose transmembrane region and POTRA domains have previously been annotated. Identified excavate candidates (**B**) conform to a Sam50-like architecture of a single N-terminal POTRA domain (≈80aa, green), and a C-terminal transmembrane region (≈300aa, blue).

While both prokaryotic and eukaryotic outer membrane insertases share a 300 aa C-terminal transmembrane domain (also annotated in literature as a bacterial surface antigen, Bac_surface_Ag, domain) corresponding to the predicted β-barrel portion of the membrane protein, the domain organization of the N-terminal region differs between prokaryotes and eukaryotes (Figure 1). This region in prokaryotes contains tandem repeats of the POTRA (polypeptide-transport-associated domain) domain, in the Omp85 family (Figure 1B), while only a single domain is present in eukaryotic proteins (Figure 1C). These differences also contribute to a size difference between these two protein families. The candidate translocases identified in this study from genome resources (Supplementary Table S1) have a common structure similar to Sam50 (Figure 1C). A minor difference in architecture is observed in the *T. vaginalis* candidate, which lacks a short N-terminal region outside the recognized POTRA domain; however, this is consistent with N-terminally shortened sequences in other hydrogenosomal proteins [14].

Supplementary Figure S1 (available at http://www.bioscirep.org/bsr/033/bsr033e084add.htm) illustrates the POTRA domain from both full-length proteins detected by HMM and EST fragments of the N-terminal region found through tBLASTn. Common motifs between eukaryotic and prokaryotic POTRA domains have previously been characterized [24,25], and the excavate sequences share several common features including N- and C-terminal patterns of repeated hydrophobic residues, and a core -G–I/V–F- motif.

### Phylogeny of putative Sam50 candidates

Full-length sequences of excavate Sam50-like proteins were aligned with iterative MAFFT [18] in addition to full-length proteins from OEP (outer envelope protein), Toc75, Omp85 and YtfM families. A consensus dendrogram constructed from Bayesian analysis and Neighbour-Joining was used to infer phylogeny of proteins within the alignment (Figure 1A). These results show that the excavate putative insertases belong to a eukaryotic group, most closely related to annotated Sam50 homologues and distinct from prokaryotic groups and Oep80/Toc75.

### Localization of T.v.Sam50 to the *Trichomonas* hydrogenosome

**Figure 2:**
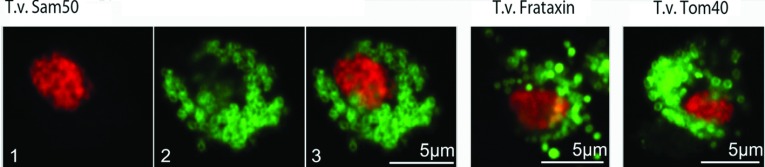
*T. vaginalis* putative membrane insertase localizes to the hydrogenosome Transformant cells containing HA-tagged T.v.Sam50, hydrogenosomal lumenal protein frataxin and hydrogenosomal membrane protein Tom40 (TVAG_450220, TrichomonasDB; or A288G1, Uniprot) were immunocomplexed with an Alexa488 tagged antibody and visualized through excitation with an argon laser (2), nuclear staining was also conducted with propidium iodide (1) and excited with a solid-state laser. Combined images (3, right) reveal that all proteins localize to similarly sized organelles consistent with the hydrogenosome, although exhibit different internal spatial localization.

An N-terminally HA-tagged Sam50 candidate (T.v.Sam50) was transformed into *T. vaginalis* strain C1 using a modified pTagVag vector. The localization of expressed protein was examined through confocal microscopy. Figure 2 shows the localization of immunodetected HA-tagged T.v.Sam50 alongside a similar C-terminally HA-tagged construct containing the lumenal hydrogenosomal protein frataxin [13] and an N-terminally HA-tagged hydrogenosomal homologue to Tom40. *Trichomonas* encodes a number of paralogous Tom40 proteins; this study employs the previously characterized paralogue THOM-C [26] also described as Tom40-3 [9], with database accessions TVAG_450220 (Trichomonas DB) or A288G1(Uniprot). All the three proteins localize to abundant, evenly distributed, sub-micrometre spherical bodies within the parasite's cytosol (Figure 2, and Supplementary Figure S2 at http://www.bioscirep.org/bsr/033/bsr033e084add.htm)]. However, subtle differences in localization are apparent, with the T.v.Sam50 protein localized to ring-like features consistent with a membrane, or a membrane peripheral localization as previously identified with T.v. Tom40, in contrast to the solid punctate signal from the lumenal protein frataxin.

### Subcellular localization of T.v.Sam50 to hydrogenosomal membranes

**Figure 3:**
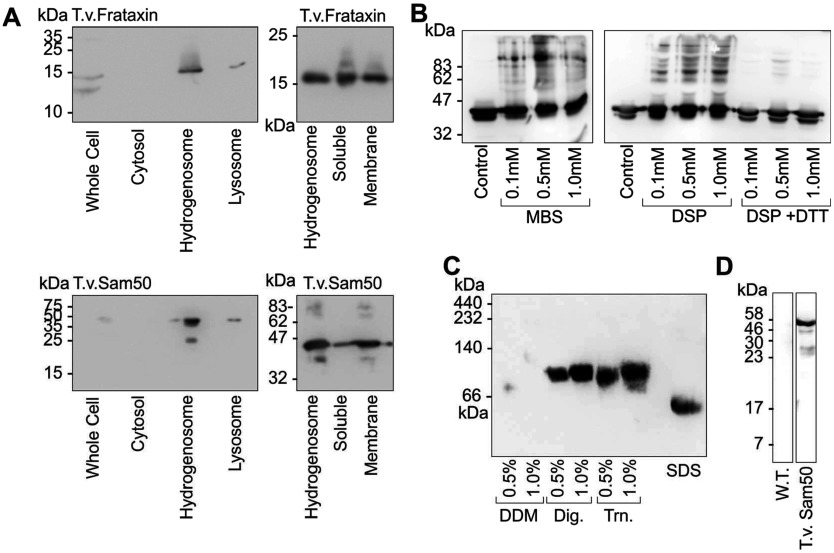
T.v.Sam50 localizes to the hydrogenosomal membrane and forms oligomeric complexes Characterization of T.v.Sam50 was investigated through molecular biology techniques. Subcellular fractionation (**A**) of whole cell lysates from T.v.Sam50, and HA tagged frataxin share a predominantly hydrogenosomal localization, and that the putative membrane protein T.v.Sam50 predominantly resides within membrane fractions obtained from purified organelles. The potential for complexes was shown through chemical crosslinking (**B**) with crosslinkers MBS and DSP. The addition of crosslinkers generates multiple discrete oligomeric complexes, which for DSP can be reversed by the addition of DTT. Detergent solubilized hydrogenosomes were also investigated for complexes by BN-PAGE (**C**) under different detergent conditions and show a shift relative to an SDS-denatured control. Triton X-100 solubilized hydrogenosomes were also subject to co-immunoprecipitation (**D**) where it was demonstrated that recombinant HA-tagged T.v.Sam50 could be recovered by immunoprecipitation. All panels are Western blots detected with anti-HA antibodies as described in the text.

Subcellular localization was further confirmed through molecular biology techniques, and subcellular fractions were obtained from transformant T.v.Sam50 expressing cultures (Figure 3A, left). Identity of the hydrogenosomal fraction was made both by visual inspection of the separated fraction (which has a classical distinctive brown hue; results not shown) and by comparison against the previously characterized hydrogenosomal proteins T.v.Tom40 and frataxin. Among the collected fractions T.v. Sam50 was most highly detected within the hydrogenosomal fractions. Further investigation with purified organelles illustrates that membrane extraction with sodium carbonate retains the majority of T.v.Sam50 to a membrane protein fraction, with a similar distribution as observed with T.v. frataxin [9,26] (Figure 3A, right). In parallel, and to rule out the possibility that the HA-tag was influencing cellular localization, we demonstrated that endogenous Sam50 was also resident in the hydrogenosomal membrane (Supplementary Figure S3 at http://www.bioscirep.org/bsr/033/bsr033e084add.htm). Thus, we have clear evidence that the T.v. Sam50 protein is a hydrogenosomal, membrane-embedded protein.

### T.v.Sam50 crosslinks to multiple discrete species within the hydrogenosome

The potential for T.v.Sam50 to form complexes was further probed through chemical crosslinking with the agents DSP and MBS (Figure 3B). Under incubation conditions of 5 mg/ml hydrogenosomal protein concentration and crosslinker concentration between 0.1 and 1.0 mM, discrete supra-molecular bands were observed, these species were found to be reversibly cleaved with DTT in DSP crosslinked samples. The presence of discrete supramolecular species suggests T.v.Sam50 could associate with a distinct complement of proteins to form oligomeric assemblies.

### T.v.Sam50 forms detergent soluble supra-molecular complexes

Further evidence for the existence of molecular complexes containing T. v. Sam50 was obtained in the absence of cross-linkers, by solubilizing purified hydrogenosomes under a variety of mild non-denaturing detergent conditions (Figure 3C). Solubilized samples were found to contain T.v.Sam50 protein, which showed an apparent size shift under BN-PAGE relative to an SDS-denatured control. These results could indicate the presence of protein complexes under these detergent conditions, although might also reflect an electrophoretic retardation resulting from the tertiary structure of T.v.SAM50, an effect also seen with other β-barrel proteins [27]. T.v.Sam50 complexes solubilized in 1% (v/v) Triton-X100 coimmunoprecipitated using a mouse monoclonal anti-HA antibody. T.v.Sam50 was found to be recovered after immunocomplexed proteins were precipitated using protein-A sepharaose, and eluted with glycine (Figure 3D), although the yield of such complexes was insufficient to allow for identification of interacting partners.

## DISCUSSION

The collected bioinformatic data for the excavate clade, and the molecular biology characterization of a putative Sam50 insertase in Trichomonas highlight aspects of the evolution of the excavate clade, and of mitochondrially derived organelles. Bioinformatic analysis of sequenced species revealed a broad distribution of Sam50 in excavates with the exception so far of any candidates in *Giardia* or *Spironucleus*. These results support previous research which also suggests that that the *Giardia* mitosome either lacks an identifiable Sam50-like insertase or employs a different protein for insertase activity [23].

This investigation rules out the presence of homologues similar to OEP80/TOC75, OMP85 and YtfM serving as an outer membrane insertase in *Giardia*. These results support a Sam50 within several groups of excavata (Euglenozoa, Heterolobosea and Parabasalids), and improving resources for *Spironucleus* and *Trimastix* would highlight the evolutionary state of the *Giardia* mitosome.

Phylogenetic and domain architecture analysis of excavate candidates reinforce their relation to other eukaryotic Sam50-like proteins, rather than related insertases from prokaryotic or plastid sources. This evidence would suggest that the candidates descend from a distinctly mitochondrial protein, and the insertases of excavata and other eukaryotes share an ancestor structurally distinct to the OMP85 protein of the mitochondrial endosymbiont.

Current knowledge about excavate Sam50 homologues is limited, work from trypanosomatid mitochondria [8] has identified the potential for excavate Sam50 to form complexes (characterized at 250–300 kDa) and functional activity with respect to the insertion and biogenesis of β-barrel preprotein. The investigation of *Trichomonas* Sam50 supports characteristics consistent with other Sam50 homologues. T.v.Sam50 was found to be localized to the hydrogenosomal membrane, and capable of forming inter and/or intra-molecular complexes, as observed for other outer membrane β-barrel insertases [28]. It is demonstrated here that T.v.Sam50 has solubilization characteristics similar to other eukaryotic Sam50 homologues, and can be purified from hydrogenosomes.

The evidence from localization and bioinformatic analysis would suggest that *T. vaginalis* was once in the possession of a mitochondria-like organelle, which contained a Sam50 protein, and while this study cannot shed light on the continued evolution of the mitochondrion to hydrogenosome, these results are consistent with a hypothesis that the outer membrane insertase complex in the hydrogenosome arose from a mitochondrial source, and not from the insertases of a secondary endosymbiont [7].

## Online data

Supplementary data
