# Supplementary material for: Analysis of the Sam50 translocase of Excavate organisms supports evolution of divergent organelles from a common endosymbiotic event
Source: Biosci Rep. 2013 Dec 3;33(6):e00084. doi: 10.1042/BSR20130049 (PMC3848468; doi:10.1042/BSR20130049)
Supplement: Supplementary data [file bsr033e084add.pdf]

Biosci. Rep. (2013) / **33** / art:e00084 / doi 10.1042/BSR20130049

OPEN ACCESS

Original Paper

Bioscience Reports

www.bioscirep.org

Bioscience Reports

SUPPLEMENTARY DATA

Analysis of the Sam50 translocase of Excavate organisms supports evolution of divergent organelles from a common endosymbiotic event

Christopher J. KAY*, Karen LAWLER* and Ian D. KERR*[1]

*School of Life Sciences, University of Nottingham Medical School, Queen's Medical Centre, Nottingham NG7 2UH, U.K.

UniPROT	EST	Organism		Aligned sequence	
Q7RK67		*Plasmodium yoelii*	2	--EIDPQQKITDVTVNLEGINKIKKRRLEFIF-------KELKKSKNIEQVFYNIAQCNNKIVNLNIFEDYPIV----KLNSLINSHIIIDYIFKEKKK	87
B3L7X3		*Plasmodium knowlesi*	3	-EKNPLSQKLTSVQVNVEGLKKLKGEKLNFLF-------EDIRKSATLEDLFLHINKCNEKIHRLNIFADVPVI----NLRSITDTQVEVKYAFREQTN	89
C5LPE8		*Perkinsus marinus*	26	-IHRVQDERTPEVRVHVKGRRRLNEALLEKFI-------GPLRMCDTYGELHDTLNRTIRDLEELNAFEDIRATVDHMPGSP--PGSADVTLEVVEKQR	125
Q4UAZ3		*Theileria annulata*	6	-YNLDVSKKLGNVRVFAKGLTKIKYSTISKDI-------EKLKDSKNLGELLTQLQQAHQSLDRLGVFKGLTTNVV--RGDQ--EGDVDVVFTFEEKPA	93
A4HIY6		*Leishmania braziliensis*	15	DLKVAMKMPIRAH-VRLVGIEKTHPDVVARDL-------ETIKRCLTIQEAVETITEIRSRWISAGIFRSVQYNFE--PTADGNQNDICVRLDVVEEKP	113
G9HQ56		*Leishmania tarentolae*	15	DLNAALKIPIRAH-VRLVGIEKTHPDVVARDL-------ETIKRCQTMQQAVEAITEIRSRWISAGIFRSVKYNFE--PTADGERNDVCVHLDVIEEKP	113
E9AE43		*Leishmania major*	15	DLSAALKMPIRAH-VRLVGIEGTHPDVVASDL-------ETIKRCRTMEEAVEAITEIRSRWIAAGIFRNVKYNFE--PTADGERNDICVRLDVVEEKP	113
A4I4P0		*Leishmania infantum*	15	DLSAALKMPIRAH-VRLVGIERTHPDVVARDL-------ETIKRCRTMQEAVEAITEIRSRWISAGIFRNVKYNFE--PTADGERNDICVRLDVIEEKP	113
E9BKZ1		*Leishmania donovani*	15	DLSAALKMPIRAH-VRLVGIERTHPDVVARDL-------ETIKRCRTMQEAVEAITEIRSRWISAGIFRNVKYNFE--PTADGERNDICVRLDVIEEKP	113
E9ALP2		*Leishmania mexicana*	15	DLSTALKMPIRAH-VRLVGIEKTHPDVVARDL-------ETIKRCRTMQEAVEAITEIRSRWISAGIFRSVKYNFE--PTADGERNDICVRLDVIEEKP	113
E7LD12		*Trypanosoma cruzi*	56	DIEKVMDLPIRTH-VRIRGIQRIHPRVISRDV-------EAIKRSRTITEITNNMSEAKSRWVQMGVFNSVNFNLE--PTYDGEANDVCVHIDVEESKP	144
G0TTA9		*Trypanosoma vivax*	16	DIKESLHLPVRAH-VRVRGIEKLHPRVISRDL-------EAIKRSRTIFDITNNMNEAKSRWVRLGIFNDVKFNLE--PTFDGAPDDVCVHIDVVESKA	104
Q580K4		*Trypanosoma brucei*	20	DIGKVLNVPIRTH-VRIRGIENIHPRVISRDV-------EAIKRSENIEDITNNMNEARTRWARMGIFSKVDFNLE--PTFHGESNDVCVHIDVVEAKP	108
	EC690752	*Jakoba libera*	33	-LHANWETPVSVEGVRVHGLQRTSPVFFRRFVSSCLGGPGALRAPLPLGSLVARMSATHEELMALEIFSDVKIG----PIAGRSGGQSAVLDILVKEKK	126
	EC784699	*Reclinomonas americana*	17	---------------NAVSANATRPDDILDFF-------------RGLTVEVNNTDAEGRLVLVDGIARARKFLAP------------DVIVDMATL--	73
	EC813295	*Seculamonas ecuadoriensis*	47	--GVIVDELLRGSGERSEGRRRGSDGRVLDTV-----VAAPVLAVLRVHPAVERQAAVRRGVRALGLVVR--------EGEELAT-NDDLKALVVDHGP	130
	EC722572	*Malawimonas jakobiformis*	45	----------------ISGLHRIRPDIVEREF-------KPVSEATSFAQLIAESERAARRLRSMGVFQSVDVLRD--EGSDPTLSRVKV---VVEEYK	115
D2VX42		*Naegleria gruberi*	16	-QPDAGRENLQLKEIFVRGVEATSESKITAAF-------QEAKKATTLNELNEAISIGLERVGKLDVLNNVNVILD-----ADDSKNVALVLDCKEKQS	101
A2DIG2		*Trichomonas vaginalis*	1	--------MSSAPEWFPTG---SLQEAIDEV---------KKLTVDDEFKEAAMKSIAEKVLQLGSIKS--------ATIDAKTGEYNVVARDGLMKT	70
Q6ATX4		*Oryza sativa*	58	-RLSSDPVGIRVHDVIIKGNAKTKEELIEAEV------AELLRAAPTVQDLLRNASIASARLRQLDVFDSVNITLD--AGPPELPGTTNVVVEVVEAAN	148
Q9SRL6		*Arabidopsis thaliana*	68	-RMRAAPVPVRVHDVIIGGNEKTKDHIIEAEV-------DAVREATTLQELLEASRVANSNLRALDIFDSVNITLD--SGPPELPGTTNVVIDVVESKS	157
Q9V784		*Drosophila melanogaster*	15	-KYDLSKISARVDRVNVSGLLRTHNDYVMRAA-------DGLFKASNFQDLMLEAMSTKSYLHELGIFKDVSVHIDVSRGADASPQGYEVTFKGNEMSR	105
Q5U3I0		*Danio rerio*	37	-QEVLENKDVVVQHVNIEGLGRTKEDYLGYEI-------SDVFTARNLVEVMRKSHEARQRLLRLGIFRDVEVVIDISEGADALPNGLDVTFEVTELKR	137

Figure S1 Alignment of the POTRA domain of selected putative Excavate OMP85 proteins
Sequences identified by HMM search of genomic data, and tBLASTn search of EST databases were aligned and the region around the POTRA domain is illustrated above. The previous work by Kutik et al. [1] identifies several domain features (indicated in grey) common between eukaryotic and prokaryotic OMP85 proteins, in this alignment additional conserved residues are indicated between the reference sequences and Excavates (green).

[1] To whom correspondence should be addressed (email ian.kerr@nottingham.ac.uk).

© 2013 The Author(s) This is an Open Access article distributed under the terms of the Creative Commons Attribution Licence (CC-BY) (http://creativecommons.org/licenses/by/3.0/) which permits unrestricted use, distribution and reproduction in any medium, provided the original work is properly cited.

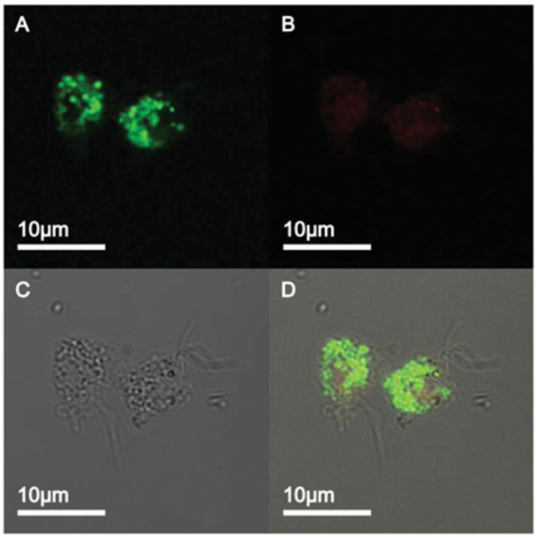

Figure S2 The hydrogenosomal marker protein frataxin was found localized to bodies within the cytosol of *Trichomonas* consistent with previous characterization of this protein
This figure illustrates the localization and distribution of an HA-tagged frataxin (**A**), alongside the distribution of the propidium iodide marker (**B**) and DIC image (**C**). A combined channels image (**D**) shows that the localization of frataxin is intra-organellar, and the cells maintain a well-defined morphology.

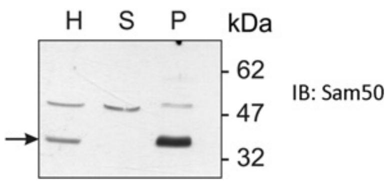

Figure S3 Immunolocalization of Sam50 to the hydrogenosome membrane
T. vaginalis strain G1 was fractionated as described and western blots probed with a rabbit polyclonal antibody to bacterially expressed and purified Sam50. The arrow indicates Sam50, with non-specific reactivity evident at higher molecular weights. Abbreviations: H, hydrogenosome fraction; S, hydrogenosome lumen fraction; P, hydrogenosome membrane fraction.

Table S1 Identities to putative Excavate Omp85 family proteins
A HMM modelled on eukaryotic SAM50 homologues was able to detect candidates from the genome data of *Trypanosoma*, *Leishmania*, *Trichomonas* and *Naegleria*, in addition the search was also able to detect putative candidates from the amoebazoan *Entamoeba*, which also possess highly degenerate metabolic organelles, the Uniprot accessions for three proteins is indicated to the left. An attempt was also included to search the NCBI EST databases of incompletely sequenced Excavates using tBLASTn, the most significant results (error of $<1.0\times10^{-4}$, region of alignment >100aa) are indicated including within which domain of the protein they were detected. Some of these ESTs have been further investigated in alignments.

(a) SAM50 HMM

UniPROT accession	Organism	E-value (HMMER)
G0TTA9	*Trypanosoma vivax*	1.60×10^{-28}
Q580K4	*Trypanosoma brucei*	1.60×10^{-24}
E7LD12	*Trypanosoma cruzi*	5.60×10^{-24}
Q4CQX2	*Trypanosoma cruzi*	2.90×10^{-23}
Q4CQ17	*Trypanosoma cruzi*	6.20×10^{-23}
F9WBB3	*Trypanosoma congolense*	7.80×10^{-19}
E9AE43	*Leishmania major*	2.70×10^{-22}
A4HIY6	*Leishmania braziliensis*	7.40×10^{-22}
E9ALP2	*Leishmania mexicana*	8.20×10^{-22}
E9BKZ1	*Leishmania donovani*	1.50×10^{-21}
A4I4P0	*Leishmania infantum*	1.50×10^{-21}
G9HQ56	*Leishmania tarentolae*	3.30×10^{-21}
A2DIG2	*Trichomonas vaginalis*	3.60×10^{-5}
B0ERN9	*Entamoeba dispar*	0.0011
C4M537	*Entamoeba histolytica*	0.0028
D2VX42	*Naegleria gruberi*	1.40×10^{-42}

(b) POTRA region

NCBI EST accession	Organism	E-value (tBLASTn)
EC690752	*Jakoba libera*	9.0×10^{-7}
EC812874	*Seculamonas ecuadoriensis*	9.0×10^{-5}
EC813295	*Seculamonas ecuadoriensis*	5.0×10^{-5}
EC716173	*Malawimonas californiana*	6.0×10^{-7}
EC722572	*Malawimonas jakobiformis*	4.0×10^{-12}
EC784699	*Reclinomonas americana*	4.0×10^{-5}
EC791291	*Reclinomonas americana*	4.0×10^{-5}

(c) Bac_surface_Ag region

NCBI EST accession	Organism	E-value (tBLASTn)
EC813725	*Seculamonas ecuadoriensis*	5.0×10^{-8}
EC630380	*Euglena longa*	1.0×10^{-13}

REFERENCE

1 Kutik, S., Stojanovski, D., Becker, L., Becker, T., Meinecke, M., Krüger, V., Prinz, C., Meisinger, C., Guiard, B., Wagner, R. et al. (2008) Dissecting membrane insertion of mitochondrial beta-barrel proteins. Cell **132**, 1011–1024

Received 8 May 2013/18 July 2013; accepted 19 July 2013
Published as Immediate Publication 23 October 2013, doi 10.1042/BSR20130049

© 2013 The Author(s) This is an Open Access article distributed under the terms of the Creative Commons Attribution Licence (CC-BY) (http://creativecommons.org/licenses/by/3.0/) which permits unrestricted use, distribution and reproduction in any medium, provided the original work is properly cited.
